# The combined effect of food additive titanium dioxide and lipopolysaccharide on mouse intestinal barrier function after chronic exposure of titanium dioxide-contained feedstuffs

**DOI:** 10.1186/s12989-021-00399-x

**Published:** 2021-02-17

**Authors:** Yongliang Zhang, Shumin Duan, Ying Liu, Yun Wang

**Affiliations:** 1 Department of Occupational and Environmental Health Sciences, School of Public Health, Peking University, Beijing, 100191 People’s Republic of China; 2 CAS Key Laboratory for Biomedical Effects of Nanomaterials and Nanosafety & CAS Center for Excellence in Nanoscience, National Center for Nanoscience and Technology of China, Beijing, 100190 People’s Republic of China

**Keywords:** Titanium dioxide nanoparticle, Lipopolysaccharide, Nutrient absorption, Gut barrier, Inflammation

## Abstract

**Objective:**

Up to 44% of particulates of food-grade titanium dioxide (TiO_2_) are in nanoscale, while the effect and combined effect of which with other substances on intestinal barrier haven’t been fully understood yet. This study is aimed to study the effect of two kinds of TiO_2_ nanoparticles (TiO_2_ NPs and TiO_2_ MPs) on intestinal barrier functions, to reveal the combined effect of TiO_2_ NPs and Lipopolysaccharide (LPS) on intestinal barrier.

**Methods:**

Male ICR mice were randomly divided into 18 groups (3 feed types * 3 exposure length * 2 LPS dosage) and were fed with normal or TiO_2_-mixed feed (containing 1% (mass fraction, w/w) TiO_2_ NPs or TiO_2_ MPs) for 1, 3, 6 months, followed by a single oral administration of 0 or 10 mg/(kg body weight) LPS. Four hours later, the transportation of TiO_2_, the intestinal barrier functions and the inflammatory response were evaluated.

**Results:**

Both TiO_2_ notably increased the intestinal villi height / crypt depth ratios after 1 and 3 months of exposure, and increased the expression of ileal tight junction proteins (ZO-1 and occludin) after 1 month of exposure. After 6 months of exposure, TiO_2_ NPs led to reduced feed consumption, TiO_2_ MPs caused spare microvilli in small intestine and elevated Ti content in the blood cells. The intestinal permeability didn’t change in both TiO_2_ exposed groups. After LPS administration, we observed altered intestinal villi height / crypt depth ratios, lowered intestinal permeability (DAO) and upregulated expression of ileal ZO-1 in both (TiO_2_ +LPS) exposed groups. There are no significant changes of ileal or serum cytokines except for a higher serum TNF-α level in LPS treated group. The antagonistic effect was found between TiO_2_ NPs and LPS, but there are complicated interactions between TiO_2_ MPs and LPS.

**Conclusion:**

Long-term intake of food additive TiO_2_ could alter the intestinal epithelial structure without influencing intestinal barrier function. Co-exposure of TiO_2_ and LPS would enhance intestinal barrier function without causing notable inflammatory responses, and there is antagonistic effect between TiO_2_ NPs and LPS. All the minor effects observed might associate with the gentle exposure method where TiO_2_ being ingested with feed.

**Supplementary Information:**

The online version contains supplementary material available at 10.1186/s12989-021-00399-x.

## Background

Titanium dioxide (TiO_2_) is an important inorganic white pigment, and is widely used as an additive in food sector, including meat, minced fish, candy, bakery, cheese, sugar, spices, and food supplements. Considering the differences in eating habits, the daily TiO_2_ intake fluctuated between 0 and 112 mg/person [1, 2]. As TiO_2_ has the highest concentrations in candy, chewing gum and chocolate, children became the high exposure crowd. In 2012, it was estimated that the intake of TiO_2_ for every UK child under the age of 10 is 2 to 3 mg/kg per day, and every adult ingests 1 mg/kg TiO_2_ a day [3]. With the development of nanotechnology, a large number of nanoscale TiO_2_ have been produced and used. Studies have shown that 17–36% [4, 5] of food grade TiO_2_ particles are in nano-size, the variation is probably caused by different manufacturing methods. It was also reported that TiO_2_ particles extracted from commercial foods have 10–44% particles in nano-size [1, 6]. These TiO_2_ NPs can be ingested with food, which will lead to direct exposure of the digestive tract to TiO_2_ nanoparticles (NPs).

The intestinal tract is the main site for water and nutrients absorption, and is also an important barrier against invasions of foreign materials. These functions mainly depend on the integrity of the intestinal epithelial cell barrier, which is composed of intestinal epithelial cells and intercellular connections. In the intact intestinal barrier epithelium, the intercellular space is sealed by the apical junction complex, including tight junction which closes gaps between cells [7–10]. The body can directly regulate the permeability of the intestinal barrier by regulating the function of tight junction which consists of a variety of proteins, like occludin, claudin, zonula occludens (ZO), and myosin light chain kinase [11–14]. Mild mucosal epithelial damage may promote foreign materials to cross intestinal mucosal epithelium, which in turn may induce T helper cell 1 (Th1) or Th2 mediated inflammatory response characterized by increased level of tumor necrosis factor (TNF), interferon-γ (IFN- γ) and interleukin-13 (IL-13), these cytokines can impact on tight junction proteins and increase tight junction permeability which will allow more bacterial products or food antigens to cross the intestinal barrier [9, 13, 15]. This feedback will amplify inflammation and eventually leads to disease. Conversely, if the foreign materials triggered the differentiation of regulatory T (Treg) cells, intestinal mucosal hemostasis will be promoted as Th-1 cell differentiation would be suppressed, in addition, IL-10 and transforming growth factor β (TGF-β) secreted by Treg cells and retinoic acid secreted by epithelial cells would enhance tight junction integrity and maintain intestinal mucosal hemostasis [15].

Acute and subchronic oral toxicity studies have shown that the bioavailability of TiO_2_ NPs in gastrointestinal tract is very low and most of the ingested TiO_2_ NPs are excreted with feces [16–19], suggesting that after being orally ingested, most of the ingested TiO_2_ NPs would move through the gastrointestinal tract, making the gastrointestinal tract one of its main target organs. In vitro studies have revealed that TiO_2_ NPs exposure can directly damage intestinal epithelial microvilli and compromise the integrity of Caco-2 cell monolayer, including the intercellular connections [20, 21]. Our earlier in vivo research [22] also found that oral exposure to TiO_2_ NPs could downregulate the plasma D-lactate level and the activity of diamine oxidase (DAO), implicating its potential to affect intestinal permeability. Inflammation is an important mechanism for toxic effects caused by nanomaterials, including TiO_2_ NPs. Nogueira et al [23] set up a 10-day oral-exposure study and found that TiO_2_ NPs induced Th1 cells dominated inflammatory response in the small intestine, the ileum showed the most sever inflammatory response among all the segments of the small intestine. Inflammatory response is associated with downregulated expression of tight junction proteins [24–28], which may further increased epithelial permeability and health risks. Though it’s unclear yet whether TiO_2_ NPs would impair intestine barrier function by inducing inflammation or not. On the other hand, it’s easy for the ingested TiO_2_ NPs to engage with other substances in gut, like lipopolysaccharide (LPS) from bacteria. Several in vitro studies [29, 30] have already indicated co-exposure of TiO_2_ NPs and LPS may trigger severer inflammatory response, suggesting the potential of TiO_2_ NPs to interact with LPS and affect gut functions, but these findings still need to be verified in vivo.

So far, there is still limited number of in vivo studies over TiO_2_ NPs influencing intestinal functions, and most of them are confined to evaluating the capability of TiO_2_ NPs to cross the intestinal mucous membrane and accumulate in or impact on other organs [16, 31, 32]. Furthermore, these in vivo studies adopted oral gavage where TiO_2_ particles were suspended in liquid medium and administrated intensively, which is not in accordance with the experience of human where TiO_2_ particles are being ingested in milder ways, like multiple food or drug intakes. In addition, the dosage is usually high and the exposure time is usually short, with a maximum exposure period up to 90 days [17, 18, 31, 33, 34]. While the public typically have a low TiO_2_ NPs dosage and a long exposure period, so, the chronic toxicity profiles of TiO_2_ NPs still need to be supplemented.

For these considerations, we simulated human exposure scenarios by mixing TiO_2_ NPs into feeds and feeding it to mice for up to 6 months, aiming at exploring the chronic effect of TiO_2_ NPs on intestinal barrier. Furthermore, we attempted to verify whether long term exposure to TiO_2_ NPs would exacerbate the impact of bacterial toxins (Lipopolysaccharides, LPS) on intestine barrier.

## Results

### Physiochemical properties of titanium dioxide

**Fig. 1:**
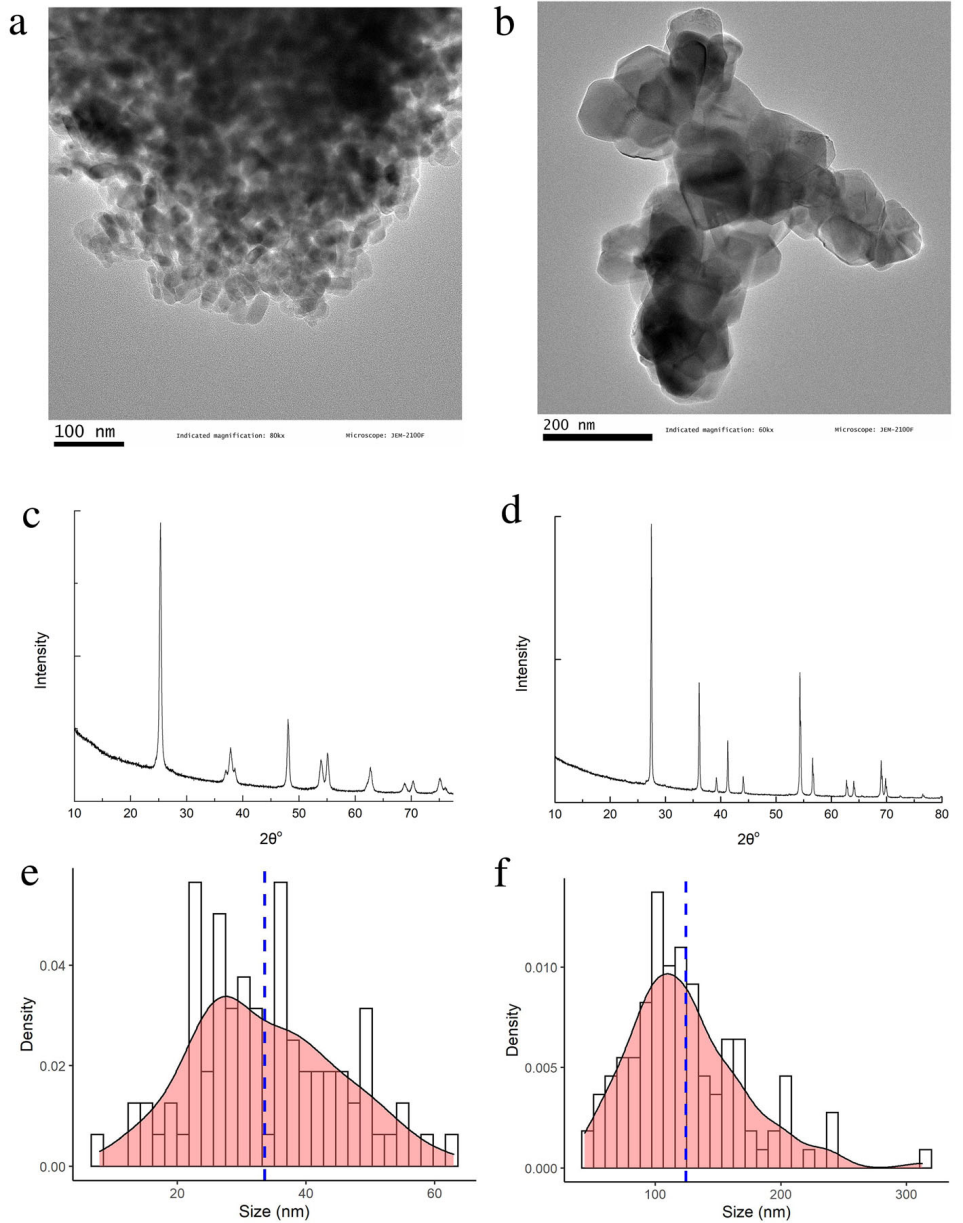
TEM images, XRD images and TEM based size distributions of TiO_2_ NPs (**a**, **c**, **e**) and TiO_2_ MPs (**b**, **d**, **f**)

As shown in Fig. 1, the two TiO_2_ nanoparticles (TiO_2_ NPs and TiO_2_ MPs) both had the nearly spherical shape. TiO_2_ NPs was in anatase form and TiO_2_ MPs was in rutile form as tested by X-ray powder diffractometry (XRD). The purities were both over 99.95% (Impurity elements were presented in the Additional file 1: Table S1). The average primary diameters of TiO_2_ NPs and TiO_2_ MPs measured by transmission electron microscopy (TEM) were (33.6 ± 11.5) nm and (124.5 ± 46.1) nm respectively, and the Brunauer-Emmett-Teller (BET)-specific surface area were 61.87 m^2^/g and 9.35 m^2^/g respectively.

According to characterization of food additive TiO_2_ (E171) purchased from Chinese and European vendors, E171 was a mixture of micron-sized particles and nano-sized particles (NPs) with anatase, rutile, or anatase/rutile-mixed form [35]. The diameter of E171 ranged from 40 to 200 nm with 10–36% particles in nanoscale [1, 5, 36], which makes it difficult to explain whether the biological effects of E171 depends on its nano-fraction, micro-fraction, or the interaction of the two fractions. However, nano-fraction has attracted more attention since nanoparticles could exhibit completely different physiochemical properties as well as different biological impacts compared to their native bulk compounds. Hence, anatase TiO_2_ NPs and rutile TiO_2_ MPs which had relatively uniform particle sizes were used in this study to represent the different crystal components of food additive TiO_2_ E171 within nanoscale and help us to understand the health risk of nano-fraction of E171 [35].

Different from previous studies that dispersed TiO_2_ in ultrapure water for exposure via oral gavage, we mixed TiO_2_ into feed and fed it to mice for exposure. To compare the characteristic changes of the TiO_2_ particles ingested via oral gavage and via mixed feed in the gastrointestinal tract, the hydrodynamic diameters, polydispersity index (PDI), and zeta potential were tested carefully when the particles and particle-mixed feeds were dispersed or digested in ultrapure water (H_2_O), artificial gastric juice (AGJ) and artificial intestinal juice (AIJ). As shown in Table 1, both TiO_2_ would aggregate into larger particles in H_2_O, AGJ and AIJ, with the biggest hydrodynamic diameters and the worst dispersion stability in the AGJ. We also found the particles in feeds aggregated into the biggest particles in AGJ but the smallest particles in AIJ after the feeds were digested in AGJ for 2 h and further in AIJ for a 2.5 h. These results suggested that TiO_2_ ingested via the two exposure routes presented different physiochemical properties in the gastrointestinal tract, which would result in different biological effects.

**Table 1:** The dispersion state of TiO_2_ particles in ultrapure water, artificial gastric/intestinal juice (AGJ/AIJ)

Material	Ultrapure water			AGJ			AIJ		
	Hydrodynamic diameter(nm)	PDI	Zeta-potential(mV)	Hydrodynamic diameter(nm)	PDI	Zeta-potential(mV)	Hydrodynamic diameter(nm)	PDI	Zeta-potential(mV)
TiO_2_ NPs ^a^	942.6±319.8	0.90±0.11	−18.37±1.04	1350±248.95	0.99±0.02	2.37±0.92	805.3±265.0	0.83±0.17	−23.67±1.68
TiO_2_ MPs ^a^	1071.9±128.7	0.95±0.08	−15.37±0.35	1497±366.38	1.00±0.00	2.15±0.99	1194.3±183.6	0.99±0.02	−13.53±0.45
TiO_2_ NPs-mixed feed	NT	NT	NT	1560.7±561.9^b^	0.97±0.05^b^	−3.17±0.26^b^	348.2±54.8^c^	0.47±0.10^c^	−6.48±1.34^c^
TiO_2_ MPs-mixed feed	NT	NT	NT	1636.4±722.8^b^	0.97±0.09^b^	−0.92±1.22^b^	670.3±286.0^c^	0.79±0.18^c^	−13.03±2.03^c^

Notes. a: Hydrodynamic diameters, PDI and zeta potential of TiO_2_ particles after being suspended in ultrapure water, AGJ or AIJ (the final test concentration is 0.5 mg/mL) respectively. b: Hydrodynamic diameters, PDI and zeta potential of particles was tested after TiO_2_ NPs or TiO_2_ MPs-mixed feed being digested in AGJ for 2 h (The final test dose is 0.25 mg/mL TiO_2_). c: Hydrodynamic diameters, PDI and zeta potential of particles was tested after TiO_2_ NPs or TiO_2_ MPs-mixed feed being digested in AGJ for 2 h followed by further digestion in AIJ for 2.5 h (The final test dose is 0.125 mg/mL TiO_2_). NT:no test; PDI: polydispersity index

### Animal behavior, body weight and feed consumption

During the animal experimental period of 6 months, only one mouse in TiO_2_ NPs exposed groups showed anal swelling on the 43th day and died of intestinal obstruction two days later, all other mice showed no abnormality.

**Fig. 2:**
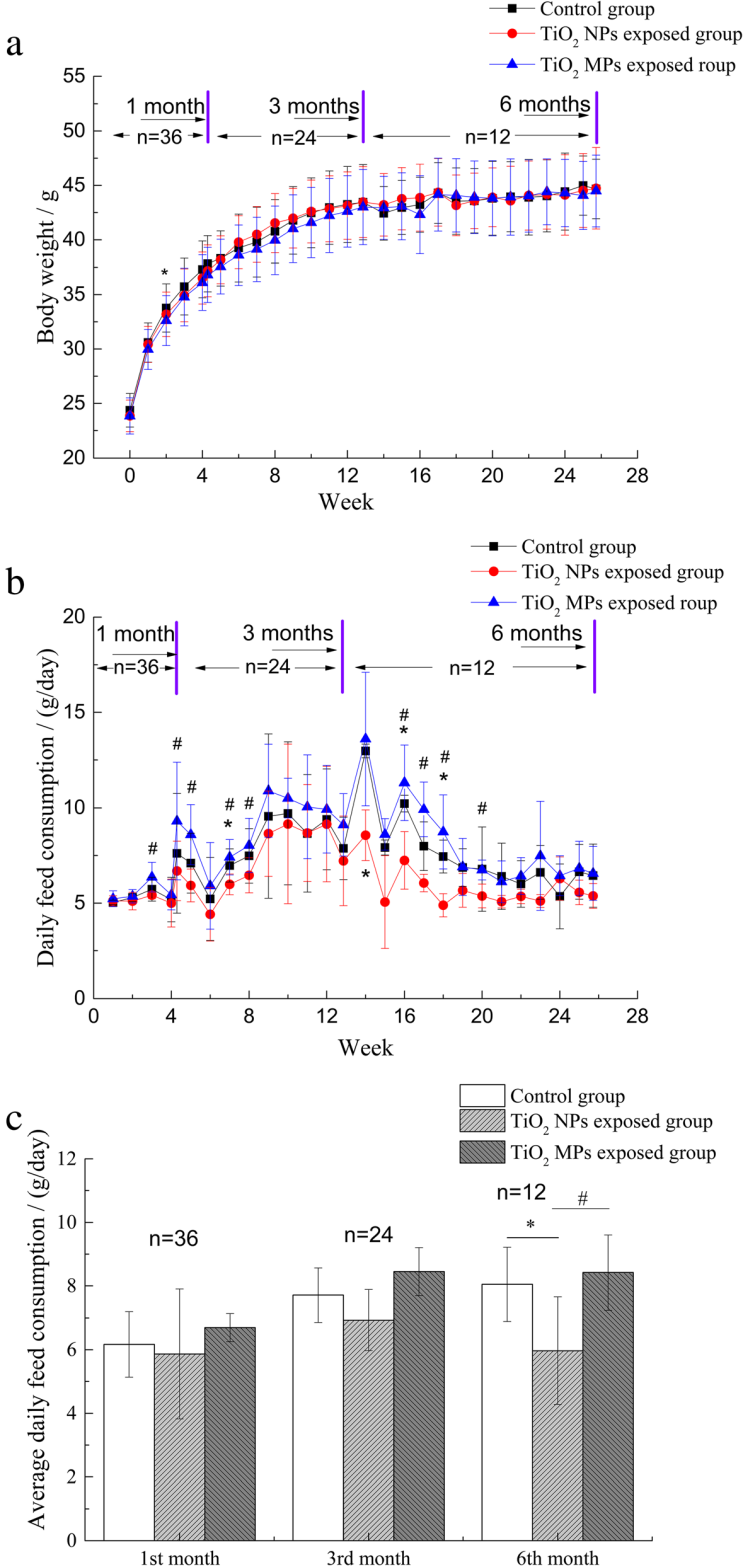
Change of body weight (**a**) and daily feed intake (**b**, **c**) in mice during exposure to TiO_2_ NPs or TiO_2_ MPs-mixed feed for 1, 3, and 6 months (mean ± SD). *: difference between TiO_2_ NP exposed group and control group is statistically significant, *p*< 0.05; #: difference between TiO_2_ NPs exposed group and TiO_2_ MPs exposed group is statistically significant, *p*< 0.05. n: number of mice per group in each period, as 4 mice were housed in one cage, n/4 data points (one data per cage) were included for sensitivity analysis

As shown in Fig. 2a, though with fluctuation, body weights of mice in either control, TiO_2_ NPs exposed group or TiO_2_ MPs exposed group continued to increase since exposure and remained stable from the 17th week onwards. No statistically significant differences in body weight were found between the control group, TiO_2_ NPs exposed group and TiO_2_ MPs exposed group during the exposure period of 6 months, except for the reduced body weight in TiO_2_ MPs exposed group by the end of the 2nd week when compared to control group.

We observed notable change of daily feed intake in TiO_2_ NPs exposed group. Comparing to control group, daily feed intake of mice in TiO_2_ NPs exposed group decreased in the 7th, 14th, 16th and 18th week, while no significant change was observed in TiO_2_ MPs exposed group (Fig. 2b). We also found that daily feed intake of mice in TiO_2_ NPs exposed group is lower than that of TiO_2_ MPs exposed group in several interval weeks. When feed up to 6 months, the average daily feed intake per mouse in TiO_2_ NPs exposed group significantly reduced compared to control group and TiO_2_ MPs exposed group (Fig. 2c).

### Bio-transport of ingested TiO_2_

**Fig. 3:**
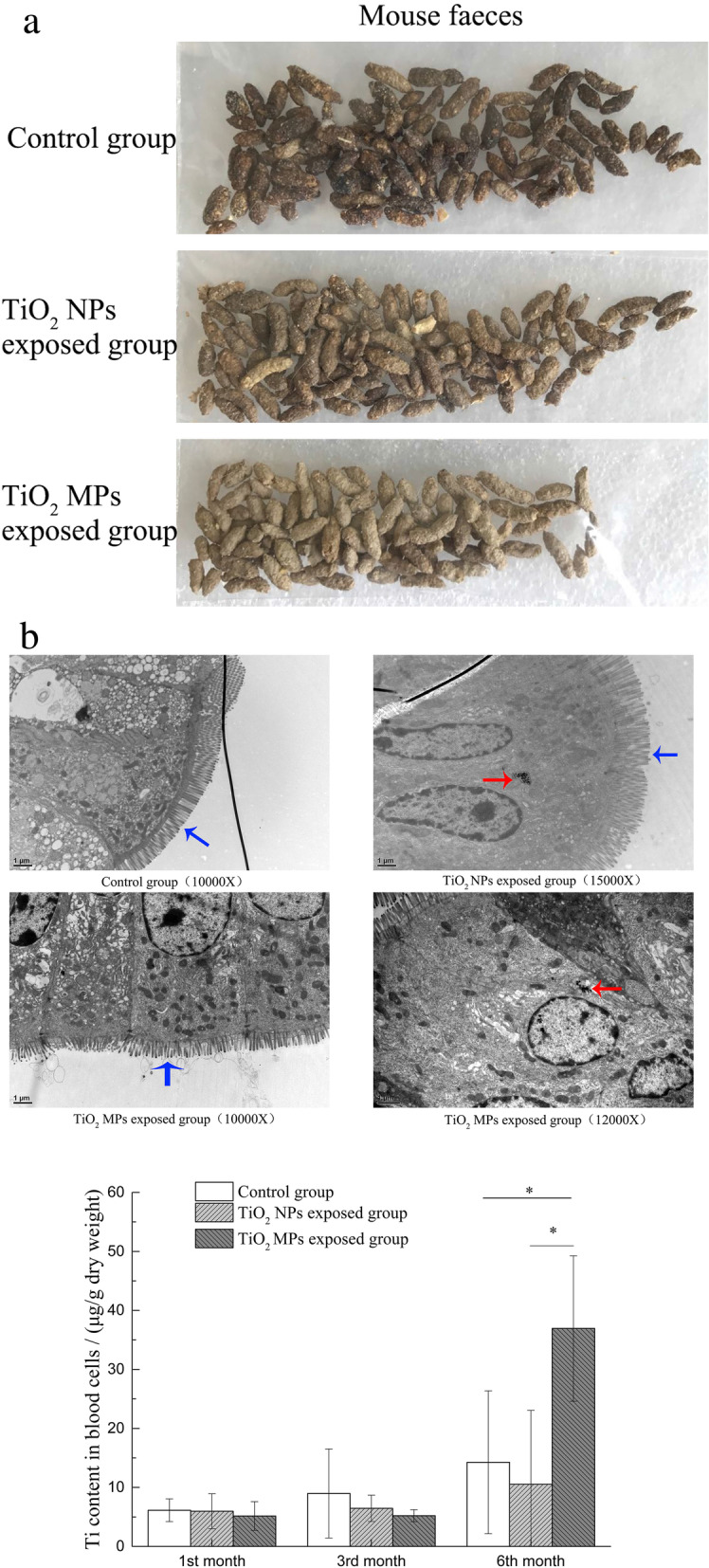
The translocation of TiO_2_ particles from the gastrointestinal tract. (**a**) Fecal images of mice fed with control feed, 1% (w/w) TiO_2_ NPs or TiO_2_ MPs mixed feed for 21 days of exposure. Similar feces were observed from day 21 onwards (from day 21 to the end of exposure). (**b**) TEM images of ileum mucosa in mice after fed with TiO_2_ NPs or TiO_2_ MPs-mixed feed for 6 months. Red arrows indicate TiO_2_ particles internalized into intracellular vesicles. Blue arrows indicate microvilli. (**c**) Ti content in mice blood cells (μg/g dry weight) after feeding with control, 1% (w/w) TiO_2_ NPs or 1% (w/w) TiO_2_ MPs-mixed feed (mean ± SD, n=5) for up to 1, 3, 6 months

During exposure, mice in both TiO_2_ exposed groups started excreting white feces from the 3rd week of exposure onwards (Fig. 3a), indicating that TiO_2_ were excreted via feces. Although TiO_2_ particles were observed in the intestinal epithelial cell cytoplasm in both TiO_2_ NPs and TiO_2_ MPs exposed groups (Fig. 3b, further introduced below), the increased Ti content in blood cells was only found in the TiO_2_ MPs exposed group after 6 months of exposure (Fig. 3c).

### Intestinal permeability

**Fig. 4:**
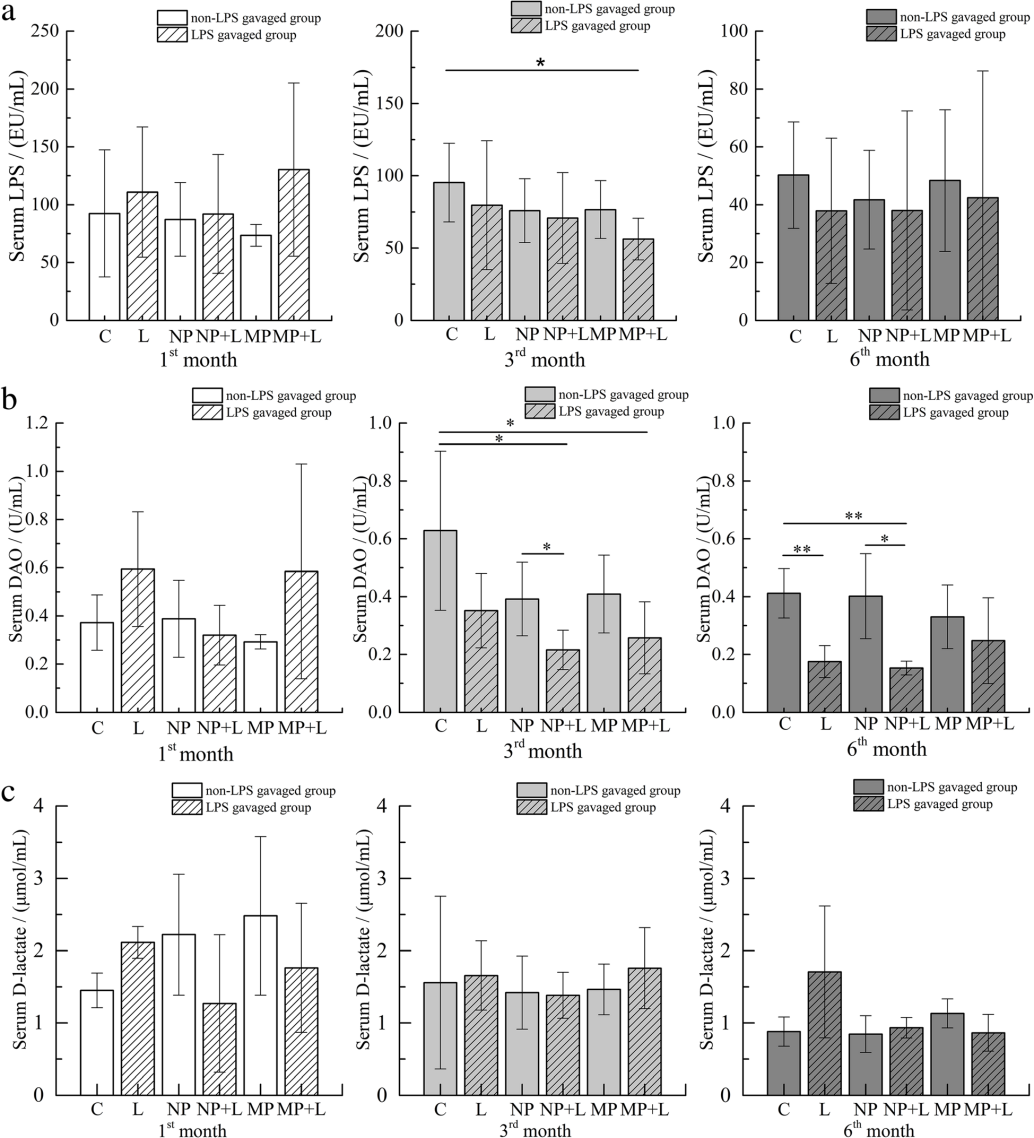
Change of intestinal permeability in mice after treatment with TiO_2_ and lipopolysaccharides (LPS). The levels of LPS (**a**), diamine oxidase (DAO) activity (**b**) and D-lactate (**c**) in mice serum after mice being fed with TiO_2_ NPs or TiO_2_ MPs-mixed feed for 1, 3, and 6 months and following gavage with LPS (mean ± SD, *n* =4). Significant differences between two groups (*p< 0.05, **p< 0.01)

Levels of serum LPS, D-lactate and DAO were detected to evaluate the permeability of the intestinal barrier. As shown in Fig. 4, significant changes were found in LPS and DAO but not in D-lactate. No differences were observed when comparing these indexes in TiO_2_ NPs or TiO_2_ MPs exposed groups with control groups. However, a significant decrease in serum DAO was found in the LPS exposed group in the 6th month (see exposure group in Table 2), lower DAO levels were found in (TiO_2_ NPs + LPS) exposed group at the 3rd and the 6th month, we also observed lower LPS and DAO levels in (TiO_2_ MPs + LPS) group at the 3rd month. The serum DAO level in (TiO_2_ NPs + LPS) exposed group was also notably lower than in TiO_2_ NPs exposed group at the 3rd and the 6th month. Through interaction analysis (Table 3), we only observed an antagonistic interaction between TiO_2_ MPs and LPS at the 6th month over serum DAO, no interaction was observed between TiO_2_ NPs and LPS over intestinal permeability.

**Table 2:** Animal grouping and treatment

Group	Treatment
C	Feed with normal feed for 1, 3, 6 months
NP	Feed with TiO_2_ NPs-mixed feed for 1, 3, 6 months
MP	Feed with TiO_2_ MPs-mixed feed for 1, 3, 6 months
L	ig 10 mg/kg LPS after feeding with the normal feed for 1, 3, 6 months
NP+L	ig 10 mg/kg LPS after feeding with the TiO_2_ NPs-mixed feed for 1, 3, 6 months
MP+L	ig 10 mg/kg LPS after feeding with the TiO_2_ MPs-mixed feed for 1, 3, 6 months

Note: ig, intragastric administration

**Table 3:** The interaction between TiO_2_ particles and LPS on mice

Interaction between	Exposure Time of TiO_2_	Parameters of									
		Intestinal permeability			Tight junction proteins		Villi height/crypt depth ratio			Inflammatory biomarkers	
		DAO	LPS	D-lactate	ZO-1	Occludin	Duodenum	Jejunum	Ileum	Serum	Ileum
TiO_2_ NPs and LPS	1 month	–	–	–	–	Ant.	Ant.	–	Ant.	IL-1β (Ant.)	–
	3 months	–	–	–	–	–	–	Ant.	Ant.	–	–
	6 months	–	–	–	–	–	–	–	–	–	–
TiO_2_ MPs and LPS	1 month	–	–	–	–	–	Ant.	Ant.	–	–	–
	3 months	–	–	–	–	–	Syn.	Syn.	–	–	–
	6 months	Ant.	–	–	–	–	–	–	–	–	–

Note: 2*2 factorial design analysis. “-“, no interaction effects, *p*> 0.05. Syn.: synergistic effect (p< 0.05), Ant.: antagonistic effect (*p*< 0.05)

Collectively, TiO_2_ NPs or TiO_2_ MPs exposure did not influence intestinal permeability, LPS stimulation following TiO_2_ exposure reduced intestinal permeability.

### Intestinal histopathological examination

**Fig. 5:**
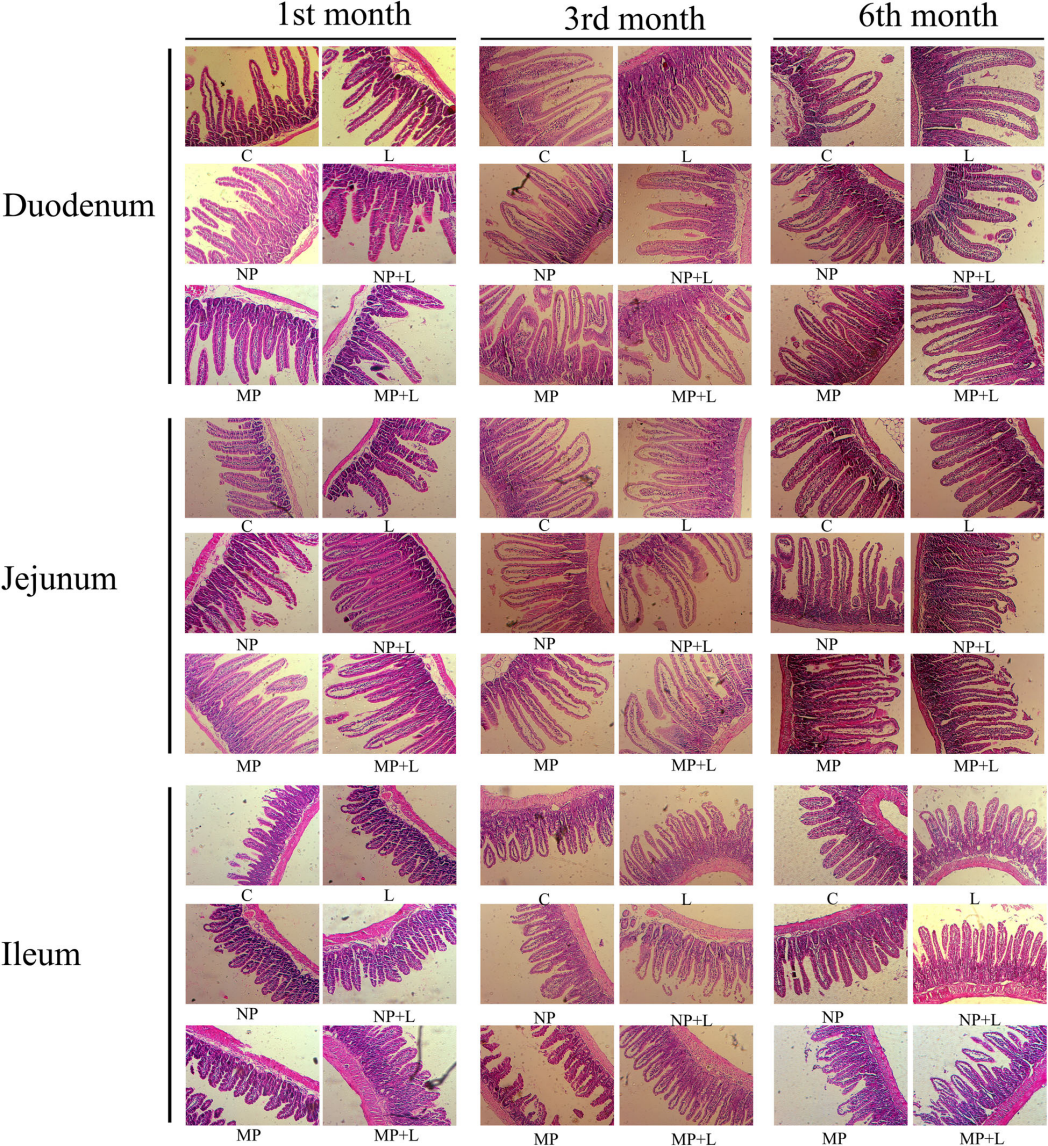
Histopathological examination of mice small intestine after treatment with TiO_2_ and lipopolysaccharides. Representative Figures are shown for each group, and three mice per group were included for histopathological assessment (*n*=3)

In control group at each time interval, intestinal villi of duodenum, jejunum and ileum are all long and intact, the intestinal cells are well lined up, structures of intestinal crypts are clear, no hyperaemia, edema or inflammatory cell infiltration were observed in all the small intestine segments. No abnormality was observed in TiO_2_ NPs exposed group or TiO_2_ MPs exposed group except for increased eosinophil in both groups at the 1st, the 3rd and the 6th month (Fig. 5). LPS stimulation didn’t cause any noticeable changes as well.

We also assessed the villi height/crypt depth ratio in different segments of the small intestine (Table 4). Compared with the control group, in the duodenum, the villi height/crypt depth ratio increased significantly after 1-month exposure to TiO_2_ NPs or TiO_2_ MPs as well as after LPS stimulation by the end of the 1st month, but the ratio decreased after LPS stimulation by the 3rd and the 6th month. In the jejunum, the villi height/crypt depth ratio increased after 1- and 3-month exposure to TiO_2_ MPs or (TiO_2_ NPs + LPS). In the ileum, the villi height/crypt depth ratio decreased after 1-month exposure to TiO_2_ NPs, (TiO_2_ MPs + LPS) and after LPS stimulation by the 1st month, but the ratio increased after 3-month exposure to TiO_2_ NPs or TiO_2_ MPs. In further interaction analysis (Table 3), we observed antagonistic interactions in villi height/crypt depth ratio between TiO_2_ NPs and LPS in the duodenum after 1-month exposure, in the jejunum after 3-months exposure, and in the ileum after 1-month and 3-months exposure. The interactions between TiO_2_ MPs and LPS over the villi height/crypt depth ratio in duodenum and jejunum were antagonistic effect after 1-month exposure but synergistic effect after 3-months exposure.

Collectively, exposure of TiO_2_ NPs and TiO_2_ MPs mostly increased the villi height/crypt depth ratio in the small intestine, but LPS stimulation mainly reduced the villi height/crypt depth ratio. Although increased jejunal villi height/crypt depth ratio presented in (TiO_2_ NPs + LPS) group, an antagonistic interaction was found between TiO_2_ NPs and LPS. Ileal villi height/crypt ratios decreased in (TiO_2_ MPs +LPS) group, the interaction between TiO_2_ MPs and LPS were antagonistic after 1-month exposure and synergistic after 3-month exposure. It should be noted that these effects on villi height/crypt depth ratios of the small intestine disappeared when the TiO_2_ exposure lasted for 6 months.

**Table 4:** Villi height/ crypt depth ratio in the small intestine after treatment with TiO2 and LPS

	Duodenum villi height/ crypt depth ratio			Jejunum villi height/ crypt depth ratio			Ileum villi height/ crypt depth ratio		
Group	1 month (n=15)	3 months (*n*=5)	6 months (n=5)	1 month (*n*=15)	3 months (*n*=5)	6 months (*n*=5)	1 month (*n*=15)	3 months (n=5)	6 months (n=15)
C	3.21±0.56	3.74±0.79	4.32±0.45	3.55±0.46	3.4±0.31	4.08±0.65	1.96±0.29	1.58±0.13	1.93±0.28
L	4.45±0.84^a^	1.95±0.63^a^	3.51±0.49^a^	3.98±0.69	3.25±0.42	3.15±0.67	1.67±0.12^a^	1.62±0.07	1.89±0.38
NP	3.85±0.70^a^	3.99±0.65	3.89±1.08	4.10±0.80	3.61±0.71	4.01±0.50	1.65±0.12^a^	2.05±0.24^a^	1.78±0.14
NP+L	3.31±0.65^b, c^	2.86±0.46^b, c^	3.62±0.57	5.18±1.44^a, b, c^	4.74±0.96^a, b^	3.78±0.24	1.78±0.21^c^	1.77±0.15	2.03±0.28
MP	4.41±0.60^a, c^	4.27±0.31	4.79±1.04	4.62±0.7^a^	5.03±0.92^a, c^	4.86±0.43^c^	1.69±0.12	1.79±1.61^a^	2.02±0.34^c^
MP+L	3.74±0.85^b, e^	3.72±0.48^b, d^	4.28±1.11	3.69±0.66^d, e^	3.63±0.44^d, e^	3.93±0.54^d^	1.41±0.14^a, b, d, e^	1.68±0.07	1.92±0.29

Note: a, b, c, d, e, f represent statistically significant differences when compared to C, L, NP, (NP + L) and MP group respectively, *P*< 0.05

### Intestinal epithelial ultrastructure and expression of tight junction proteins

Among the 6-months-fed groups, the ultrastructures of ileal epithelial of control group, TiO_2_ NPs exposed group and TiO_2_ MPs exposed group were assessed (Fig. 3b). Some spherical shaped particles with diameter of (48.5 ± 13.9) nm and (84.9 ± 16.0) nm were observed in the epithelial cell cytoplasm in TiO_2_ NPs and TiO_2_ MPs exposed groups respectively (Fig. 3b), which parallel the shape and size of TiO_2_ NPs and TiO_2_ MPs being used in this study therefore it’s high likely that these observed particles are TiO_2_ particles. Comparing to control group, height and density of microvilli were reduced in TiO_2_ MPs exposed group while not in TiO_2_ NPs exposed group. No other ultrastructural changes were observed in both TiO_2_ exposed group.

**Fig. 6:**
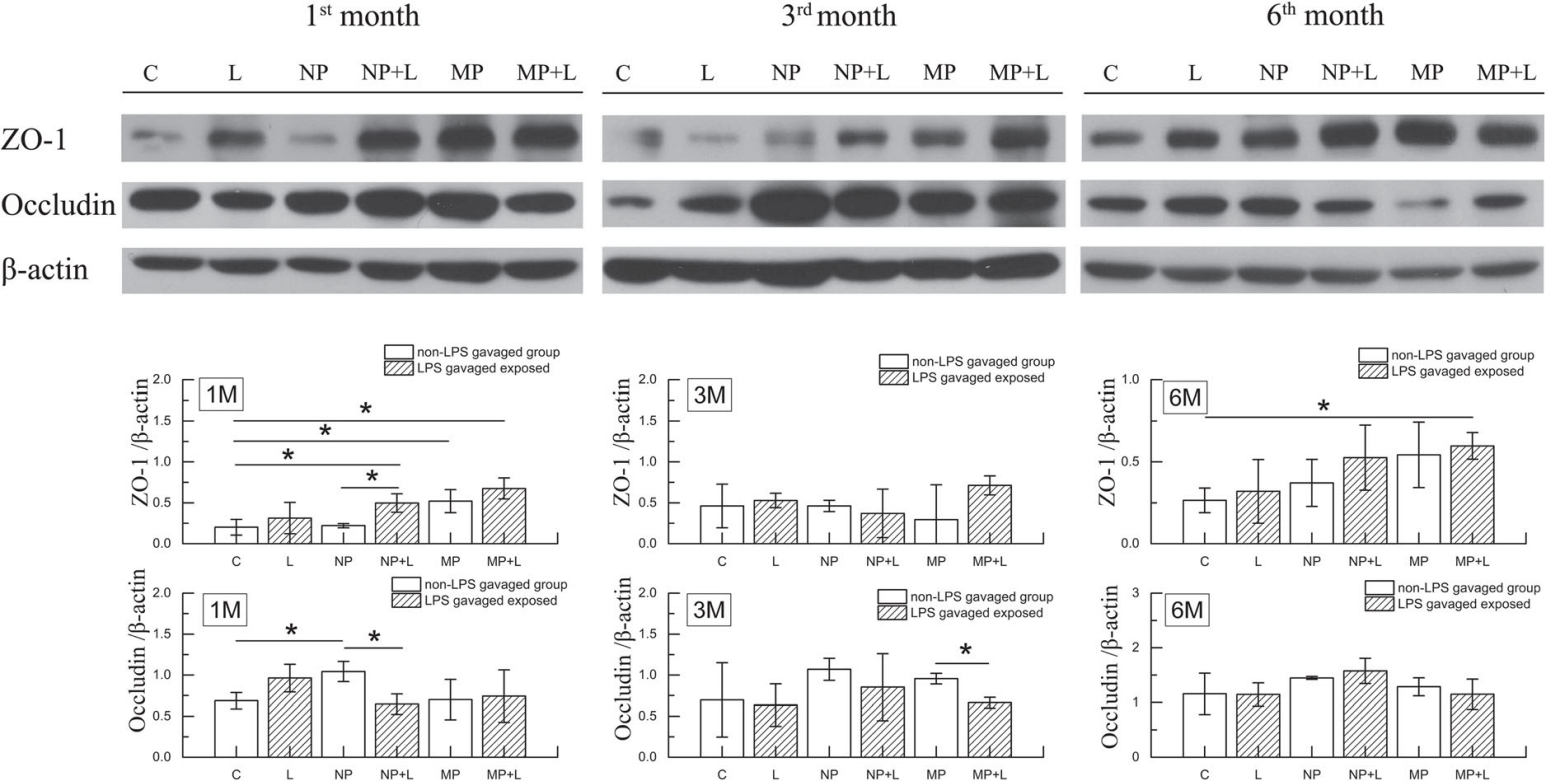
Tight junction proteins in mice ileum tissues. Levels of ZO-1 and occludin in mice ileum tissues after mice being fed with control feed, TiO_2_ NPs-mixed feed or TiO_2_ MPs-mixed feed for 1, 3, 6 months and following gavage with or without 10 mg/kg LPS (mean ± SD, *n* =3). Significant differences between two groups (* *P*< 0.05). 1 M: 1 month; 3 M: 3 months; 6 M: 6 months

We examined expression of tight junction proteins in ileal epithelial. As indicated in Fig. 6, 1-month exposure to TiO_2_ NPs notably increased occludin expression. Exposure to TiO_2_ MPs, (TiO_2_ NPs + LPS) and (TiO_2_ MPs + LPS) upregulated expression of ZO-1 at the 1st month, and (TiO_2_ MPs + LPS) exposure continued to upregulate ileal ZO-1 level significantly at the 6th month. We observed an antagonistic effect between TiO_2_ NPs and LPS over the expression of ileal occludin by the 1st month, however, such interaction disappeared by the 3rd and 6th month (Table 3).

### Inflammatory biomarker in ileum and serum

**Fig. 7:**
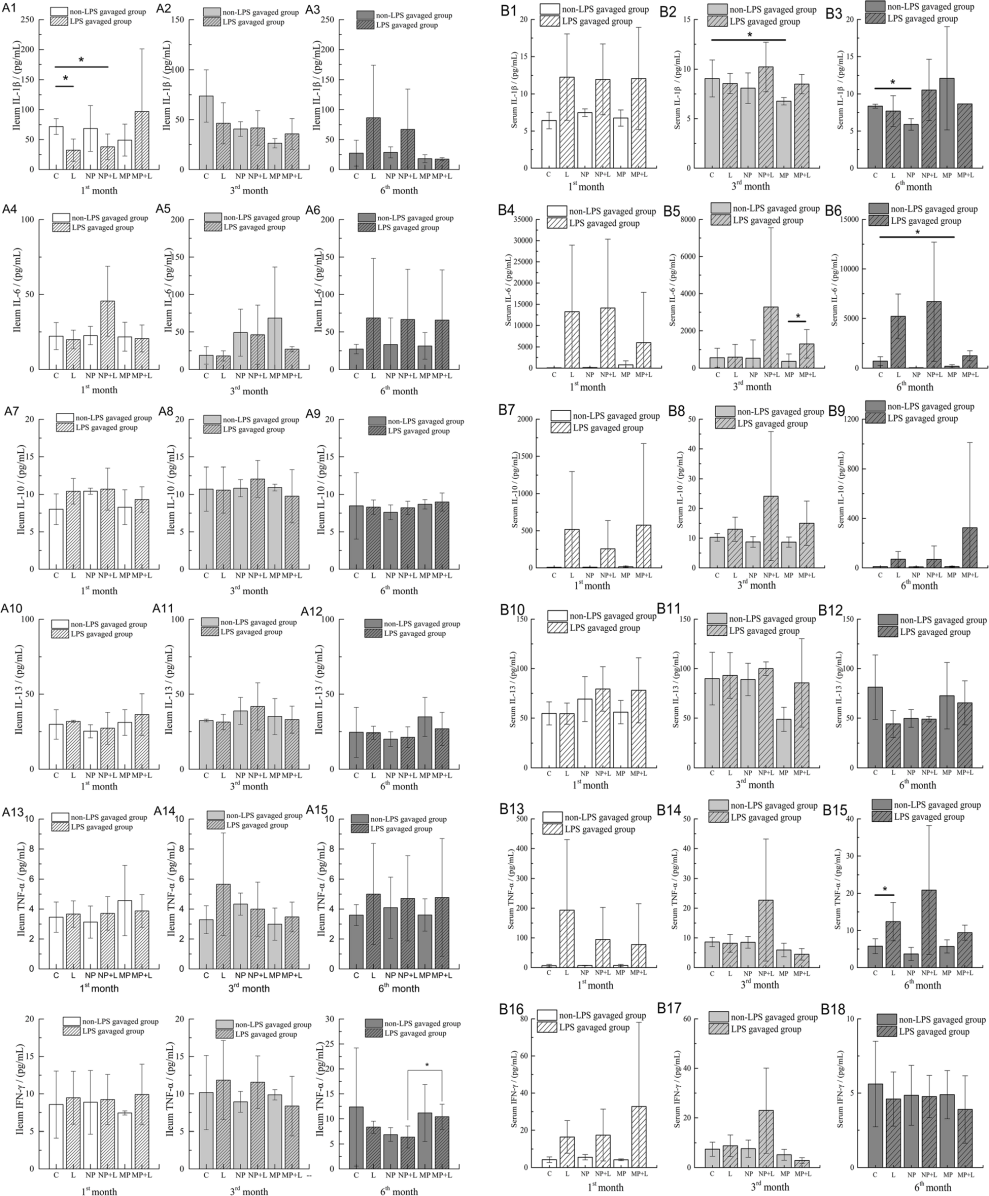
Inflammatory cytokines in mice ileum tissues and serum. Levels of IL-1β, IL-6, IL-10, IL-13, IFN-γ, TNF-α in mice ileum(A1-A18) and serum (B1-B18) after being fed with TiO_2_ NPs or TiO_2_ MPs-mixed feed for 1, 3, 6 months and following gavage with or without LPS (mean ± SD, *n* =4). Significant differences between two groups (*p< 0.05)

In ileum tissues, levels of IL-1β, IL-6, IL-10, IL-13, TNF-α and IFN-γ in both TiO_2_ NPs and TiO_2_ MPs exposed groups remained similar as control group (Fig. 7 A1-A18). After stimulation with LPS, ileal IL-1β levels in LPS group and (TiO_2_ NPs + LPS) exposed group at the 1st month were significantly lower than in control group, while no difference was observed between LPS and (TiO_2_ NPs + LPS) group. We didn’t observe any interaction between both TiO_2_ and LPS over these ileal cytokine levels (Table 3).

Serum inflammatory biomarkers were also not seen change notably (Fig. 7 B1-B18). Comparing to control group, serum IL-1β level was significantly lower in TiO_2_ MPs exposed group at the 3rd month and in TiO_2_ NPs exposed group at the 6th month, serum IL-6 level in TiO_2_ MPs exposed group notably dropped at the 6th month. After stimulation with LPS, LPS group displayed higher serum TNF-α levels than control group at the 6th month, but the cytokine levels of (TiO_2_ NPs + LPS) and (TiO_2_ MPs + LPS) groups were still similar to control group at all time points. By the 6th month, we observed an antagonistic interaction of TiO_2_ NPs and LPS over serum IL-1β levels (Table 3).

## Discussion

The highlights of this study are that TiO_2_ was mixed into feed and fed to mice for up to 6 months for exposure. Such exposure method was subtle and avoided any potential stimuli associated with oral gavage that has been used in most in vivo studies. In the meanwhile, the exposure method is more comparable to actual exposure that human experiences, where TiO_2_ particles are often been ingested along with food. Moreover, rodents are nocturnal and most activities happens during the night, including feeding, our exposure method guaranteed the TiO_2_ were ingested via daily activities, other exposure methods like oral gavage which often happens during the daytime fail to capture this. Hence, the research is valuable for evaluating the safety of food additive TiO_2_. Moreover, this is the first study to extend in vivo exposure of TiO_2_ particles to 6 months and reveal the minor biological impacts of TiO_2_ following chronic exposure by studying its translocation from intestine, impacting on intestinal barrier, pro-inflammation potency and its potential to interact with LPS in gut.

### Translocation of TiO_2_ NPs and TiO_2_ MPs from gut and animal behavior

In this study, ingesting TiO_2_ NPs-mixed or TiO_2_ MPs-mixed feed for 6 months did not influence body weight of the mice, but TiO_2_ NPs exposure notably decreased the feed intake. The possible explanation is that the exposure time is not long enough for the decreased feed intake influence body weight or the physical activity of mice were reduced after TiO_2_ exposure. It’s a pity were cannot verify as we have no activity data available. The low feed intake might lead to malnutrition and increased health risks in the long run, and more attention should be paid to its long-term impact on nutrition imbalance in further studies. Our previous study identified TiO_2_ NPs of small sizes could affect nutrient absorption and metabolism by inducing intestinal epithelial injury, with amino acids more susceptible than metal elements and glucose [37]. However, after 6-months of exposure to TiO_2_ in this study, no notable histopathological changes were found in the small intestine of mice, including the intestinal villi height/crypt depth ratio. It seems the intestinal nutrient absorption wasn’t increased by TiO_2_ NPs exposure. Hence, the low feed intake of TiO_2_ NPs exposed group might result in energy and nutrient deficiency, leading to malnutrition and other diseases. More researches are still needed for verification in the future.

As frequently reported, TiO_2_ particles had a very low bioavailability in both in vitro and in vivo studies [16, 18, 38, 39]. In both TiO_2_ NPs exposed group and TiO_2_ MPs exposed group, the Ti contents in blood cells were found to be low and are of similar concentration as control group, except for the increased Ti content at the 6th month in TiO_2_ MPs exposed group. And the Ti content also didn’t increased significantly in liver, kidney and spleen [40]. These results suggest the bioavailability of TiO_2_ was low and most of the TiO_2_ particles might be excreted with feces, which are in accordance with the excretion of white feces we observed. TiO_2_ particles were white pigment and the feces were easily whitened by these TiO_2_ particles when they are excreted with feces.

It’s well known that the particles with smaller size could be transported through the biological barriers easier than these with larger sizes, hence the low bioavailability could be partially explained by the agglomeration of TiO_2_ particles in the gastrointestinal tract as shown in Table 1. Once being ingested, TiO_2_ particles will move through various digestive juices, including saliva, gastric and intestinal juices. The local biomolecules within these digestive juices, feed components as well as intestinal flora products like LPS may coat onto the surface of TiO_2_ particles and form protein corona, which may substantially change physiochemical properties of TiO_2_ particles and influence its bioavailability [41, 42]. Sinnecker [43] also reported that luminal gut-constituents may both attenuate and augment the adherence of nanoparticles to cell surface, the process of which is particle size-dependent and interacting biomolecular dependent. In addition, as reported in a recent research [44], the limited Ti translocation could also be explained by the presence of intestinal mucus which can trap food-grade TiO_2_ particles.

### Effect of TiO_2_ NPs and TiO_2_ MPs on intestinal barrier function

Intestinal barrier function mainly relies on the integrity of the intestinal epithelial cell barrier, which is composed of intestinal epithelial cells and intercellular tight junctions [15]. Thus, we checked the intestinal epithelial histological structure and ultrastructure, the expression of tight junction proteins and the intestinal permeability in this study.

Based on the observations (as summarized in Table 5), both TiO_2_ increased villi height/crypt depth ratio in the small intestine at the 1 and 3 months without causing histological injuries. Sparse and short microvilli were found in TiO_2_ MPs exposed group at the 6th months. Studies [45–47] have shown that a decreased villi height/crypt depth ratio or a reduced villi surface area is considered deleterious for digestion and absorption and could result in retarded growth, conversely, an increased villi height-to-crypt depth ratio as well as an increased villi surface area could promote the intestinal digestion-absorption function. The sparse and short microvilli would reduce the villi surface area and result in decreased intestinal digestive and absorptive capacity. As microvilli structure remain unaffected in TiO_2_ NPs exposed group, it may indicate that TiO_2_ NPs increased intestinal digestion and absorption area by increasing villi height/crypt ratio, which may help explain the reduced feed consumption as discussed above. Similar results were reported by Ammendolia et al [48] where TiO_2_ NPs increased jejunal villi height/crypt ratio without damaging intestinal mucosa epithelium. In TiO_2_ MPs exposed group, though increased villi height/crypt depth ratios were observed at the 1 and 3 months, severely reduced microvilli height and density in intestinal columnar epithelium were observed at the 6 months. It suggested long time exposure to TiO_2_ MPs would cause intestinal injury, which would contribute to the increased Ti content in blood cells after 6 months exposure to TiO_2_ MPs since more particles would translocate through the damaged intestinal epithelial. Moreover, there is one study [49] showed that exposure to food-grade TiO_2_ (E171) for 100 days in rats triggered low-grade inflammation and initiated preneoplastic lesions in colon featuring increased number of aberrant crypts, and increased number of large aberrant crypt foci at the colonic mucosal surface. Although it remains unclear whether the increased villi height/crypt depth ratio we observe might also associate with the health risks of intestinal villus hyperplasia and carcinogenesis, more attention should be paid to the long-term effects of TiO_2_ exposure along with these changes.

**Table 5:** Notable changes of biological parameters in mice after treatment with TiO_2_ and LPS

Group	1 month	3 months	6 months
L	Duodenal villi height/ crypt depth ratio ↑ Ileal villi height/ crypt depth ratio ↓ Ileal IL-1β ↓	Duodenal villi height/ crypt depth ratio ↓	Duodenal villi height/ crypt depth ratio ↓ Serum DAO ↓ Serum TNF-α ↑
NP	Duodenal villi height/ crypt depth ratio ↑ Ileal villi height/ crypt depth ratio ↓ Ileal Occludin ↑	Ileal villi height/ crypt depth ratio ↑	Food intake ↓ Serum IL-1β ↓
NP+L	Jejunal villi height/ crypt depth ratio ↑ Ileal ZO-1 ↑	Jejunal villi height/ crypt depth ratio ↑ Serum DAO ↓	Serum DAO ↓ Ileal IL-1β ↓
MP	Duodenal villi height/ crypt depth ratio ↑ Jejunal villi height/ crypt depth ratio ↑ Ileal ZO-1 ↑	Jejunal villi height/ crypt depth ratio ↑ Ileal villi height/ crypt depth ratio ↑ Serum IL-1β ↓	Ileal microvilli ↓, Blood Ti ↑ Serum IL-6 ↓
MP+L	Ileal villi height/ crypt depth ratio ↓ Ileal ZO-1 ↑	Serum LPS ↓, Serum DAO ↓	Ileal ZO-1 ↑

Tight junction plays an important role in maintaining the integrity of intestinal epithelial barrier and intestinal permeability. A recent in vitro study [50] reported that TiO_2_ NPs upregulated ZO-1, occludin and claudin-2 expression in a shape- and time-dependent manner in an in vitro model (Caco-2/HT29) of the intestinal barrier. In the current study, increased ZO-1 and occludin are only observed in the 1st month, which make it difficult for us to conclude our results parallel the in vitro findings, such uncertainly is further supported by the absence of reduced intestinal permeability (no notable changes in serum DAO activity, LPS and D-lactate levels suggest no notable change of intestinal permeability) since upregulated tight junction protein would lead to decreased intestinal permeability. It is also inconsistent with our earlier research [22] where we observed declined serum DAO activity and D-lactate content after continuous gavage of 200 mg/kg TiO_2_ NPs (TEM measured size: 75 ± 15 nm; BET specific surface area: 63.95 m^2^/g) to adult SD rats for 30 days, which indicates decreased intestinal mucosal permeability. These differences may originate from the differences in animal species, exposure methods, exposure time length and the physiochemical properties of the TiO_2_ particles used.

### Inflammatory response induced by TiO_2_ NPs and TiO_2_ MPs

The intestinal immunity is an important part of systemic immunity and regulates the function of intestinal epithelial tight junction. In the present study, we analyzed five proinflammatory cytokines (IL-1β, IL-6, IL-13, TNF-α, and IFN-γ) and one anti-inflammatory cytokine (IL-10). Briefly, IL-1β, TNF-α, IFN-γ can impact on MLCK and eventually increase tight junction permeability. IL-6 is capable of inducing immune cell differentiation and plays a proinflammatory role which includes promoting T cells to differentiate into Th17 cells. IL-17, secreted by Th17 cells, and IL-13 can both upregulate claudin-2 concentration, which in turn increases intercellular pore channel permeability, thus increasing the permeability of the intestinal barrier. Anti-inflammatory cytokine IL-10 however inhibits the differentiation of Th1 cells, enabling it to play an anti-inflammatory role in the intestinal tract, thereby maintaining the homeostasis of barrier permeability.

Exposure to TiO_2_ NPs or TiO_2_ MPs for up to 1, 3, 6 months has not increased serum nor ileal cytokine levels, suggesting that TiO_2_ NPs or TiO_2_ MPs exposure did not cause notable inflammation. Nogueira et al [23] tested the potential of TiO_2_ to induce intestinal inflammation in Bl57/6 mice, they found that TiO_2_ NPs can increase cytokine IL - 4, IL - 12, IL - 23, TNF-α, IFN-γ, TGF-β levels in duodenum, jejunum and ileum, of which the ileum showed the highest level of cytokine. These different observations might be explained from several aspects. First, we used different TiO_2_ particles, the differences in physiochemical properties may result in different affinity to biomolecules and feed components, which may lead to different surface coating of these particle and different biological effects. Second, the mild reactions found in this study might attribute to the exposure method we used which avoided addition stress accompanied by gavage, and won’t lead to intensive TiO_2_ exposure in just seconds. Third, the dosage and the length of exposure were different. Last, the animal model we used in current study, the outbred ICR mice, is less sensitive to subtle stimuli, which is also an important reason for the different observations.

### Combined effect of TiO2 NPs and TiO2 MPs with LPS

After LPS stimulation, we observed increased expression of tight junction protein ZO-1 and declined serum DAO activity in (TiO_2_ NPs + LPS) exposed group and (TiO_2_ MPs + LPS) exposed group when compared to the TiO_2_ NPs exposed group and TiO_2_ MPs exposed group respectively, implying that co-exposure of TiO_2_ and LPS reduced the permeability of the intestinal barrier. The intestinal villi height/crypt depth ratio was also found after co-exposure of TiP_2_ and LPS. However, cytokines were not seen notably increased in (TiO_2_ NPs + LPS) and (TiO_2_ + LPS) treatment groups. The antagonistic effects were mostly found between TiO_2_ NPs and LPS.

The interaction between TiO_2_ and LPS have been reported in several studies. Riedle S et al [51] reported that the cell viability of bone marrow-derived macrophages was unaffected when TiO_2_ particles were applied after LPS exposure, but TiO_2_ particles could augmented LPS induced inflammation, Bianchi et al [52] found that compared to sole-exposure of LPS or TiO_2_ NPs, strengthened and more persistent inflammation in RAW264.7 cells were observed when LPS adsorbed onto nano-TiO_2_ protein corona. It is clear that these in vitro studies indicated synergistic effects between TiO_2_ NPs and LPS. Our recent work [53] found that TiO_2_ NPs could modify gut microbiota community structure and mitigate TNBS induced colitis. These contradictory findings may be well caused by the differences between in vitro and in vivo system, the different findings may also lie in the differences between physiochemical properties of TiO_2_ particle as well as the differences in dosage. In addition, TiO_2_ NPs are usually applied together with LPS in in vitro studies, while in the current study, mice were fasted for 6 h before LPS administration, and as mice is nocturnal rodent, LPS was administered during the non-active phase of the mice. For more clarity, more researches are still needed.

## Conclusion

This study found that short-term and long-term ingesting TiO_2_ NPs and TiO_2_ MPs-mixed feed would alter intestinal villi structure without impairing intestinal barrier function, however, co-exposure of TiO_2_ NPs or TiO_2_ MPs and LPs would enhance intestinal barrier function without causing notable inflammatory responses. In addition, TiO_2_ NPs showed antagonistic effect with LPS over intestinal villi height/crypt depth ratio. The gentle exposure method (i.e. feeding TiO_2_ mixed feeds) might have contributed to the mild chronic biological effects of TiO_2_ NPs observed in the current study. Since ingested TiO_2_ via mixed feed and via oral gavage could present different physiochemical properties in gastrointestinal tract which would in turn result in different biological effects, and as ingesting TiO_2_ with feed represents the exposure route of human being, the current research is very valuable for evaluating the safety of food additive TiO_2_ and more attention should be paid to exploring the biological effects of TiO_2_ NPs under realistic exposure conditions. Considering that TiO_2_ is a suspected carcinogen and might associate with the increased risk for the formation of colonic carcinoma, more researches are needed for clarifying whether the histological changes of intestinal villi and crypts associate with hyperplasia and carcinogenesis or not. In addition, there is a large number of bacteria exist in the intestine, among which Gram-negative bacteria produce LPS, the continuous effect of the combined exposure of TiO_2_ and LPS should attract attention.

## Methods

### Physiochemical characterization of TiO_2_ particles

Two food-grade TiO_2_ particles were purchased from Shanghai Yunfu Nanotechnology Co. Ltd., China. The primary size and shape of both TiO_2_ particles were determined using a transmission electron microscopy (TEM, JEM-2100F, JEOL, Japan). The crystal structures of both TiO_2_ were assessed with an X-ray powder diffractometry (XRD, X’Pert Pro, PANalytical). The specific surface areas were assessed based on Brunauer–Emmett–Teller (BET) method (Autosorb-iQ2-MP, Malvern Panalytical). The purity and impurities were analyzed with an inductively coupled plasma mass spectrometry (ICP-MS, Thermo Elemental X7, Thermo Electron Corporation).

Artificial gastric juice (AGJ) and artificial intestinal juice (AIJ) were prepared as described in our earlier study [22]. Briefly, the AGJ (pH=1.2) was prepared with 10 g/L pepsin (3800 units/mg) and 45 mmol/L HCl. The AIJ (pH=6.8) was constituted by 10 g/L trypsin and 6.8 g/L KH_2_PO_4_. Both TiO_2_ particles were suspended in ultrapure water, AGJ or AIJ respectively and were ultrasonicated for 30 min before measuring its hydrodynamic diameter, polydispersity index values (PDI) and Zeta-potential using a ZetaSizer Nano ZS90 (Malvern Instruments Ltd., Malvern, UK).

### Feed preparation and characterization

The commercial pellet diet and two kinds of TiO_2_-mixed feeds were supplied by Beijing Ke Ao Xie Li Food Co. Ltd., China. The TiO_2_-mixed feeds were produced by mixing 1% (mass fraction) TiO_2_ NPs or TiO_2_ MPs into the commercial pellet diet. This dosage was selected based on the maximum usage of TiO_2_ allowed in food, where both the U.S. Food & Drug Administration and the national food safety criteria of China (GB 2760–2014) have regulated TiO_2_ usage in food should be no more than 1% of the total weight.

TiO_2_ NPs or TiO_2_ MPs-mixed feed was digested in AGJ for 2 h firstly, and then moved into AIJ for further digestion of 2.5 h. The above experiment was conducted on a horizontal shaker to mimic feed digestion in vivo. After digestion, the suspensions were collected and ultrasonicated for 30 min to break up aggregates. The particle hydrodynamic diameter, PDI and Zeta-potential were assessed using ZetaSizer Nano ZS90.

### Animal treatment

The healthy male ICR mice of 3-week old were bred and supplied by the Department of Laboratory Animal Science, Peking University Health Science Center. The animals were fed a sterilized commercial pellet diet and deionized water ad libitum, and were housed in plastic cages in a 20 ±2 °C and 50–70% relative humidity room with a 12:12 h light-dark cycle. The animal experiments were carried out in accordance with the Guiding Principles in the Use of Animals in Toxicology adopted by Society of Toxicology and the European Union Directive 2010/63/EU for animal experiments and received approval from the Peking University Institutional Review Board (LA2019216).

After five days of acclimation, the 108 mice were randomly divided into 18 groups, 6 mice per group. As shown in Table 2, the mice were given normal feed, TiO_2_ NPs-mixed feed or TiO_2_ MPs-mixed feed respectively for up to 1, 3, 6 months, fasted for 6 h and followed by intragastric administration (ig) of 10 mg/(kg body weight) lipopolysaccharides (LPS, E.coli O111:B4, Sigma Aldrich) or same amount of deionized water. Additional LPS administration was performed to stimulate a flora disruption in the intestine and to investigate whether TiO_2_ exposure would alter the ability of the intestinal barrier to resist LPS invasion. Four hours later, the animals were anesthetized by ether, blood samples were collected from the eye artery by removing the eyeball quickly, the animals were then sacrificed. Sera were harvested by centrifuging blood samples at 3000 rpm for 10 min at 4 °C and the blood cells were kept for analyzing titanium (Ti) content. The tissues and organs including the small intestine were excised and stored in − 80 °C. During the whole experiment, four mice were housed together in a same cage, the animal behaviors, symptoms and mortality were recorded daily. The body weights and feed intake were measured weekly, and daily feed intake per mouse for each week was calculated. For calculating feed intake for longer period (like 1, 3, 6 months we reported), the daily feed intake is calculated as cumulative feed intake of the cage within the period being divided by the number of mice in the cage and the number of days of the period. For sensitivity analysis, each cage represents a data point of four mice, therefore, for the 1st month, we have 27 cages available (108 mice), representing 9 daily feed intake per group (9 cage * 3 types of feed). For the 3rd month, we have 18 cages (6 cages *3 types of feed), and 9 cages for the 6th month (3 cages*3 types of feed). The equations are shown below.

$$ \mathrm{Daily}\ \mathrm{feed}\ \mathrm{intake}\ \mathrm{for}\ \mathrm{each}\ \mathrm{week}=\mathrm{weekly}\ \mathrm{feed}\ \mathrm{intake}\ \mathrm{per}\ \mathrm{cage}/\left(7\ast \mathrm{number}\ \mathrm{of}\ \mathrm{mice}\right) $$

The daily feed intake per mouse for each period (1, 3, 6 months) was calculated as:

$$ \mathrm{Daily}\ \mathrm{feed}\ \mathrm{intake}=\mathrm{accumulative}\ \mathrm{feed}\ \mathrm{intake}\ \mathrm{per}\ \mathrm{cage}/\left(\mathrm{number}\ \mathrm{of}\ \mathrm{days}\ast \mathrm{number}\ \mathrm{of}\ \mathrm{mice}\right) $$

### Intestinal permeability assessment

The permeability of the intestinal barrier was evaluated by detecting the levels of lipopolysaccharides (LPS) and D-lactate and the activity of diamine oxidase (DAO) in serum. Four samples from each group were randomly chosen for these tests. The LPS content was measured using the ToxinSensor™ Chromogenic LAL Endotoxin Assay Kit (GenScript, USA). The D-lactate level was determined by the colorimetric method according to the manufacturer’s protocol (D-lactate Assay Kit, BioVision, USA). The DAO activity was assessed by the reaction of cadaverine dihydrochloride (Sigma, USA) as described in our previous work [22].

### Histopathological and TEM observation of gut tissues

For pathological assessment, all histopathological examinations were performed following a standard laboratory procedure. The intestinal tissues (three samples per group) were opened longitudinally and fixed in 10% formalin and embedded in paraffin blocks, from which 5 μm thick samples were sliced and placed onto glass slides. The slices were stained with hematoxylin and eosin (HE), an optical microscope was used to observe and take pictures. The identity of the slides was blinded to the pathologist. Five intact villi were randomly chosen from each slide if available, the villi height and the crypt depth were measured in duodenum, jejunum and ileum to calculate the ratio of the villi height to the crypt depth, resulting in 15 intact villi being examined per group from the three slides. In case of intact villi are insufficient, five intact villi were counted for the group from the three slides.

For TEM observation, the ileal mucosa samples from control, TiO_2_ NPs and TiO_2_ MPs group (one sample per group by the 6th month) were cut into small pieces (surface area = 1*1 mm^2^) and immediately fixed in 2.5% glutaraldehyde (pH=7.4) overnight. Then the samples were treated according to general protocols for TEM examination. The ultra-thin sections (70–100 nm) were stained with lead citrate and uranyl acetate and then were examined using a transmission electron microscopy (TEM, JEM-1400, JEOL, Japan).

### Titanium content analysis

The blood cells were taken out and thawed (five samples per group).All the samples were freeze-dried in a freeze dryer (FD-1, Beijing Detianyou Technology Development Co., Ltd) with apparatus at − 50 °C and a pressure of 1.0 Pa for 24 h, these samples weighed between 0.03–0.18 g after drying. The freeze-dried samples were digested in 2 mL nitric acid and 0.5 ml H_2_O_2_ overnight. After adding 1 mL HF, the mixed solutions were heated at about 160 °C using high-pressure reaction container in an oven chamber until the samples were completely digested. Then, the solutions were heated at 120 °C to remove the remaining nitric acid until the solutions were colorless and clear. At last, the remaining solutions were diluted to 5 mL with 2% nitric acid and then were loaded onto an ICP-MS (NexION 300D, PerkinElmer) to analyze Ti concentration in the samples. The detection limit of Ti was 1.1 μg/g. Concentrations of Ti element were expressed as milligrams per gram dried weight.

### Western blot analysis

The ileum tissue samples (three samples per group) were lysed in radioimmunoprecipitation assay buffer (RIPA buffer) supplemented with a cocktail of protease inhibitors. Then the proteins were collected after centrifugation and quantified using a Bradford protein assay kit (Beyotime Biotechnology, China). Equal amount of protein was denatured and separated on 7–12% SDS-PAGE gels and then transferred to nitrocellulose membrane (Merckmillipore). The membranes were blocked with 5% skimmed milk, subsequently incubated with primary antibodies against ZO-1 (1:2000, Abcam), occludin (1:20000, Abcam), and β-actin (1:30000) overnight at 4 °C, and then incubated with secondary antibody for 2 h at room temperature. The protein bands were detected by Western Blotting Luminol Reagent (Absin Bioscience Inc., China) and recorded on Kodak films (Eastman Kodak Company, US). Relative band densities of the various proteins were measured from scanned films using Image J Software.

### Inflammatory cytokine analysis

The concentrations of six cytokine biomarkers in sera and the ileum tissues (four samples per group) were analyzed using Milliplex Map Kit (Cat. No. MTH17MAG-47 K, Merck Millipore, USA) following the manufacturer’s instructions. Briefly, the ileum tissues were homogenized in PBS buffer containing the protease inhibitors, then the supernatant was obtained after centrifugation. The tissue supernatants and sera samples, buffers, and cytokine standards were added into 96-well assay plates and incubated overnight at 4 °C with fluorescently labeled antibodies-coated beads which can capture IL-1β, IL-6, IL-10, IL-13, TNF-α, and IFN-γ. After further incubation with biotin-labelled detection antibodies and streptavidin-phycoerythrin conjugate, the beads were detected on a MAGPIX® multiplexing instrument (Luminex Corporation, USA). The xPONENT 4.1 software was used for data acquisition, with the calibration curve for each cytokine generated with a five-parameter logistic fit. The detection limits for the cytokine assays were 1 pg/mL.

### Statistical analysis

SPSS 20.0 was used to carry out the statistical analysis. Data exhibited a normal distribution according to the K-S test were expressed as mean ± standard deviation (SD). Independent-samples T test was used to assess the significant difference between two experimental groups. One-way variance (ANOVA) with LSD-t or Dunnett’s T3 tests was applied to evaluate the statistical significance of the differences between the experimental groups and the controls. The interaction between TiO_2_ and LPS were analyzed based on 2 × 2 factorial design analysis. A *p* value less than 0.05 was considered statistically significant.

## Supplementary Information

**Additional file 1 Table S1**. Impurity elements of TiO_2_ NPs and TiO_2_ MPs (μg/g)

## Data Availability

The datasets used and/or analyzed during the current study are available from the corresponding author on reasonable request.

## Abbreviations

TEM: transmission electron microscopy
XRD: X-ray powder diffractometry
BET: Brunauer-Emmett-Teller
ICP-MS: inductively coupled plasma mass spectrometry
AGJ: artificial gastric juice
AIJ: artificial intestinal juice
PDI: polydispersity index value
LPS: lipopolysaccharides
DAO: diamine oxidase
HE: hematoxylin and eosin
