# Supplementary material for: The combined effect of food additive titanium dioxide and lipopolysaccharide on mouse intestinal barrier function after chronic exposure of titanium dioxide-contained feedstuffs
Source: Part Fibre Toxicol. 2021 Feb 17;18:8. doi: 10.1186/s12989-021-00399-x (PMC7887831; doi:10.1186/s12989-021-00399-x)
Supplement: Supplementary file 1 — Additional file 1 Table S1. Impurity elements of TiO2 NPs and TiO2 MPs (μg/g) [file 12989_2021_399_MOESM1_ESM.docx]

Additional files

Table S1. Impurity elements of TiO_2_ NPs and TiO_2_ MPs (μg/g)

| Materials | B | Al | V | Cr | Mn | Fe | Co | Ni | As | Se | Mo | Sn | Sb | Pb | Hg |
| --- | --- | --- | --- | --- | --- | --- | --- | --- | --- | --- | --- | --- | --- | --- | --- |
| TiO_2_ NPs | 15.5 | 63.9 | 6.6 | 2.5 | 4.4 | ND | 0.02 | ND | ND | 96.8 | 2.5 | 5.1 | 1.0 | 15.4 | 0.9 |
| TiO_2_ MPs | 21.2 | 71.2 | 5.1 | 27.4 | 4.4 | ND | 0.04 | ND | ND | 95.4 | 0.2 | 11.9 | 0.7 | 18.3 | 1.6 |

Note: ND represented the concentration was below the detection limit. B, boron; Al, aluminum; V, vanadium; Cr, chromium; Mn, manganese; Fe, iron; Co, cobalt; Ni, nickel; As, arsenic; Se, selenium; Mo, molybdenum; Sn, tin; Sb, antimony; Pb, lead; Hg, mercury.
